# Single nucleotide polymorphisms of *ABCB1* gene and response to etanercept treatment in patients with ankylosing spondylitis in a Chinese Han population

**DOI:** 10.1097/MD.0000000000005929

**Published:** 2017-02-03

**Authors:** Rui-Jian Yan, Ting-Ting Lou, Yi-Fang Wu, Wei-Shan Chen

**Affiliations:** a Department of Orthopedic Surgery, the Second Affiliated Hospital, School of Medicine, Zhejiang University; b Department of Pharmacy, Tongde Hospital of Zhejiang Province, Health Bureau of Zhejiang Province; c Department of Surgery, Zhejiang University Hospital, Zhejiang University, Hangzhou, P.R. China.

**Keywords:** *ABCB1*, ankylosing spondylitis, ASAS score, BASDAI score, efficacy, etanercept, genetics, polymorphism

## Abstract

**Background:**

Etanercept was highly recommended for patients with ankylosing spondylitis (AS), as its efficacy has been confirmed in AS, while genetic polymorphisms, by affecting drug metabolism or drug receptor, lead to interindividual variability in drug disposition and efficacy. Therefore, this study aims to investigate whether ABCB1 gene polymorphisms can predict therapeutic response to etanercept in patients with AS.

**Methods:**

A total of 185 patients with AS in our hospital were recruited into our study from December 2012 to May 2015. The frequency distributions of genotype and allele of rs2032582, rs1128503, and rs1045642 were detected by polymerase chain reaction (PCR) and electrophoresis verification enzyme products method. AS patients received etanercept treatment for 12 weeks, followed by this would be evaluated by the bath AS disease activity index (BASDAI) score improvement and the assessment of spondyloArthritis international society 20/50/70 (ASAS20/50/70) score improvements to explore the relationship between genotype of *ABCB1* gene polymorphisms and therapeutic response to etanercept in patients with AS.

**Results:**

After 12 weeks, the BASDAI score mean improvement value of rs2032582 A/A genotype was 2.87 ± 0.52. The ratios of patients with rs2032582 A/A genotype reaching the BASDAI50 and ASAS20 evaluation criteria were 64.29% and 92.86%, respectively. The results indicated that efficacy of etanercept was promoted in rs2032582 A/A genotype. The BASDAI score mean improvement value of rs1128503 C/C genotype was 2.79 ± 0.54 after 12 weeks. The ratios of patients with rs1128503 C/C genotype reaching the BASDAI50 and ASAS20 evaluation criteria were 66.67% and 93.94%, respectively. The results indicated that efficacy of etanercept was promoted in rs1128503 C/C genotype. However, no significant associations were observed between rs1045642 and therapeutic response to etanercept in AS patients.

**Conclusion:**

*ABCB1* gene rs2032582 and rs1128503 polymorphisms may be associated with the efficacy of etanercept in AS patients. *ABCB1* gene polymorphisms can act as biological indicators of etanercept efficacy.

## 1 Introduction

Ankylosing spondylitis (AS) is a chronic inflammatory disorder affecting the spine, the entheses, as well as the peripheral joints.^[1]^ The inflammatory phase of AS shares similarities with rheumatoid arthritis, such as high levels of osteoclast activity and pro-inflammatory cytokine production.^[2]^ The incidence group of AS is mainly seen in males and its onset is common in early adulthood or late adolescence.^[3]^ On the contrary, the prevalence of AS in Europe, North America, and China is estimated at 0.03 to 1.8%.^[4,5]^ Currently, its treatment just focused on the combination of physical exercise, nonsteroidal anti-inflammatory and surgery therapy, but the effects were not effective in some cases due to unclear cause of AS.^[6,7]^ Etanercept was highly recommended for AS patients, as its efficacy has been confirmed in AS.^[8,9]^ But several pharmacogenetics and pharmacogenomics studies supported that genetic polymorphism may exert influences on drug metabolism and drug targets or drug receptor, leading to interindividual variability in drug disposition and efficacy.^[10,11]^

*ABCB1* is a drug transporter protein expressed on the endothelial cells of the blood–brain barrier as well as the epithelial cells of the intestine.^[12]^*ABCB1* gene, also known as multidrug resistance gene (*MDR1*), is located in the 7q21. It encodes P-glycoprotein and comprises a core promoter region and 28 exons. Importantly, the rs2032582 (G2677T/A), rsl045642 (C3435T), and rsll28503 (C1236T) are the common and functional mutation position of the *ABCB1* genetic polymorphism.^[13–15]^ In addition, Huang et al^[16]^ reported that *ABCB1* genetic polymorphisms might be associated with SSRIs treatment response in the Chinese Han depression population. Subsequently, Ozbey et al^[17]^ also confirmed that the *ABCB1* gene polymorphism can be a biological indicator of the main depression in a Turkish population. Seo et al^[18]^ demonstrated that the *ABCB1* genetic polymorphisms (rs2032582, rsl045642, rsll28503) influence the response to antiepileptic drugs in Japanese epilepsy patients. However, the function of *ABCB1* genetic polymorphisms on etanercept treatment efficacy in AS patients has not yet to be elucidated. Therefore, our study aims to investigate the relationships between *ABCB1* genetic polymorphisms and etanercept efficacy in patient with AS to reveal AS etiology and provide a potential approach for AS therapy.

## 2 Methods

### 2.1 Ethics statement

This study was approved by the Ethics Committee of the Second Affiliated Hospital, School of Medicine, Zhejiang University and obtained all patients signed informed consent, in accordance with the Declaration of Helsinki.

### 2.2 Sample collection

Our experiments were performed on 185 AS patients (128 males and 57 female with age ranging from 18 to 70 years; mean age: 37.38 ± 6.24 years) in our hospital from December 2012 to May 2015. Diagnosis of AS patients was based on the AS Standards of American Rheumatism Association in 1984,^[19]^ which showed the characteristics as follows: lower back pain that lasted for more than 3 months, mitigated after doing exercise, but no significant relief observed during the break; lumbar scoliosis; limited front and rear mobility, reduced chest expansion compared with the normal group; II-III level bilateral sacroiliitis; II-IV unilateral sacroiliitis. Exclusion criteria included patients who had been conducted any drugs or hormone therapy within 5 months before recruitment; patients suffering from cardiovascular, cerebrovascular, diabetes, cirrhosis, hepatitis, and other diseases; patients with other thromboembolic diseases; severe liver and kidney dysfunction; pregnancy; patients receiving organ transplantation; patients who gender and age-related information was not complete.

### 2.3 Single nucleotide polymorphism (SNP) sequencing

In this study, the Chinese Han population genome data in HapMap was adopted. In addition, the 3 main methods applied as follows: ① literature review method; ② looking Tag-SNP; ③ finding functional mutation sites of *ABCB1* gene by FAST SNP. Consequently, the polymorphic loci of rs2032582, rs1128503 and rs1045642 were to be detected in our study.

### 2.4 The detection of polymorphic loci

A total of 10 mL venipuncture whole blood specimens were obtained from all patients, added to the tubes containing the anticoagulant sodium citrate, shaken well and put in the refrigerator at 4°C for backup, and then the DNA in peripheral blood were extracted in 1 week. Polymerase chain reactions (PCR) primers (Shanghai Sangon Biological Engineering Company, Shanghai, China) were designed by Primer Premier 5.0 software. Subsequently, the length of the primer sequences was demonstrated in Table 1. PCR reaction system was 50 μL, among which there were 1 μL template, 1 μL forward and reverse primers, respectively, 10 mmol/L1 μL dNTP, 5 μL Taq Buffer, 5 μL 25 mmol/L MgCl2, 0.5 μL Taq enzyme, 35.5 μL water. PCR was carried out in GeneAmP PCR system 9700 (Hai Pudi biotechnology Co. Ltd., Shanghai, China) under amplification condition by touchdown PCR: 94°C for 2 minutes, 94°C for 30 seconds, 63°C for 1 minute, 72°C for 1 minute; 35 cycles. PCR amplification products were obtained 1.5% agarose gel electrophoresis, followed by purifying to extraction kit (Shanghai Sangon Biological Engineering Company, Shanghai, China). The marked Fasta sequences of rs2032582, rs1128503, and rs104564 polymorphism were downloaded from dbSNP of NCBI. In DNAMAN, the marked restriction enzyme sites of different genotypes in nucleotide sequences of 3 polymorphisms were detected to determine the enzyme of *ABCB1* gene polymorphism and then conduct PCR detection.

**Table 1:**
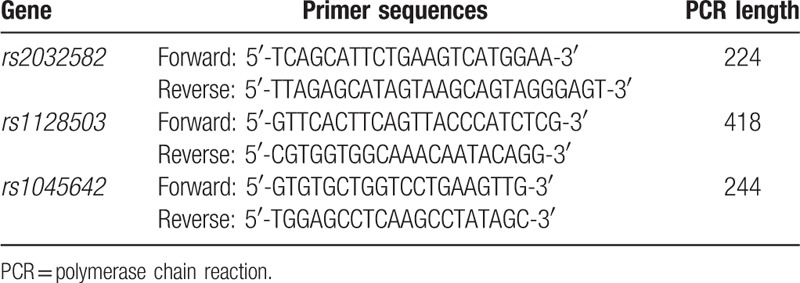
Primer sequences of *ABCB1* gene polymorphisms.

### 2.5 Therapeutic regimen

The AS patients, who had been performed genotyping, would receive etanercept (CITIC Pharmaceutical Co., Ltd., batch number 20051228). The treatment methods were as follows: subcutaneous injection of etanercept (50 mg) for once a week until 12 weeks. After 12 weeks management, the indicators of AS patients would be compared with those of origins. According to the response criteria recommended by the AS assessment working group (the assessment of spondyloArthritis international society, ASAS) to evaluate the clinical response of patients,^[20]^ 4 items were included: the total visual analogue scale (VAS) score of patients; rachialgia: the VAS score of night pain and total pain; the Bath Ankylosing Spondylitis Functional Index (BASFI); inflammatory reaction in the average VAS score of the last 2 items about the morning stiffness (MS) of the Bath Ankylosing Spondylitis Disease Activity Index (BASDAI), at least 3 items were more than 20% of BASDAI score improvement and the absolute value of VAS score improved at least 1 point, although there were no deterioration between the items unreached 20% of BASDAI score improvements and the baseline. BASDAI was used to evaluate the disease activity status of AS patients, while the disease activity status was divided into the following 5 items: A. fatigue; B. rachialgia; C. joint pain; D. enthesopathy; inflammation of the spine = 0.5 (E. MS degree + F. MS time). Each score was the VAS score of patients’ self-evaluation. Subsequently, the proportion of ASAS20, ASAS50, and ASAS70, which reached the systematic standards of ASAS, and that of more than 20% of BASDAI score improvement, 20% ∼ 50% BASDAI score improvement as well as over 50% BASDAI score improvement, were all calculated.

### 2.6 Data analysis

Data were analyzed applying the statistical package for the social sciences (SPSS) version 21.0 (SPSS Inc., Chicago, IL). Quantities data were shown as mean ± standard deviation, while counting data were represented by the number of cases or percentage. The direct counting method was applied to calculated genotype and allele frequencies and allele and genotype were compared by X^2^ test. The observation values were statistically described. Improvement ratio and percentage were inspected by X^2^ test. The BASDAI score improvement of AS patients with different genotype carriers were compared before and after treatment, where the normal distribution (*t* test for normal distribution and rank sum test for non-normal distribution) was examined at the beginning. *P* < 0.05 refers to significant test level.

## 3 Results

### 3.1 Electrophoresis of PCR product

**Figure 1:**
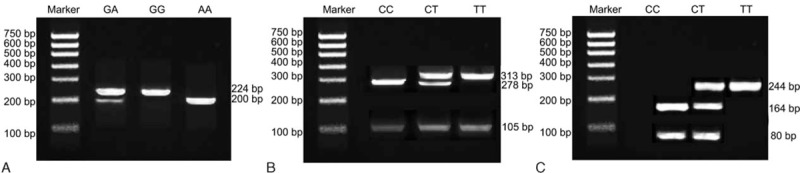
The electrophoresis of the PCR products of rs2032582, rs1128503, and rs1045642 SNPs of *ABCB1* gene. Note: A, rs2032582; B, rs1128503; C, rs1045642.

The amplified fragment length of rs2032582 was 224 bp. PCR products of mutant homozygote A/A, wild homozygote G/G, and heterozygote G/A were observed with an RsaI restriction enzyme site. Two fragments of 200 and 24 bp were presented in mutant homozygote A/A, while 3 fragments of 224, 200, and 24 bp were displayed in heterozygote G/A, and only 1 fragment of 224 bp appeared in the wild homozygote G/G (24 bp was not displayed in electrophoresis due to its small size) (Fig. 1A). Furthermore, the amplified fragment length of rs1128503 was 418 bp. PCR products of wild homozygote C/C had 2 BsuRI enzyme recognition sites. Thus, 3 fragments of 278, 105, and 35 bp appeared in wild homozygote C/C. Meanwhile, only 313 and 105 bp were detected in mutant homozygote T/T due to lack of a BsuRI restriction enzyme site. Subsequently, there were 313, 278, 105, and 35 bp in mutant heterozygote (35 bp was not showed in electrophoresis due to its small size) (Fig. 1B). The amplified fragment length of rs1045642 was 244 bp. PCR products of wild homozygote C/C were observed with an Mbol restriction enzyme site and the products were 164 and 80 bp. Only 244 bp were presented in mutant homozygote T/T due to lack of Mbol restriction enzyme site. And, there were 244, 164, and 80 bp in mutant heterozygote C/T (Fig. 1C).

### 3.2 ABCB1 polymorphisms

Among 185 cases of AS patients, there were G/G genotype (108 cases), G/A genotype (63 cases), and A/A genotype (14 cases) at rs2032582 with the frequency of 58.38%, 34.05%, and 7.57%, respectively, and 75.41% and 24.59% for G and A allele. Moreover, at rs1128503, 33 cases with C/C genotypes, 87 with C/T genotypes, 65 with T/T genotypes followed by the frequency of 17.84%, 47.03%, and 35.14% respectively, and C and T allele showed 41.36% and 58.64%. At rs1045642, the frequency of C/C genotype (74 cases), C/T genotypes (82 cases), and T/T genotype (29 cases) were 40.00%, 44.32%, and 15.68%, respectively. The Hardy–Weinberg genetic equilibrium test indicated that each genotype and allele frequency have reached representative genetic equilibrium (Table 2).

**Table 2:**
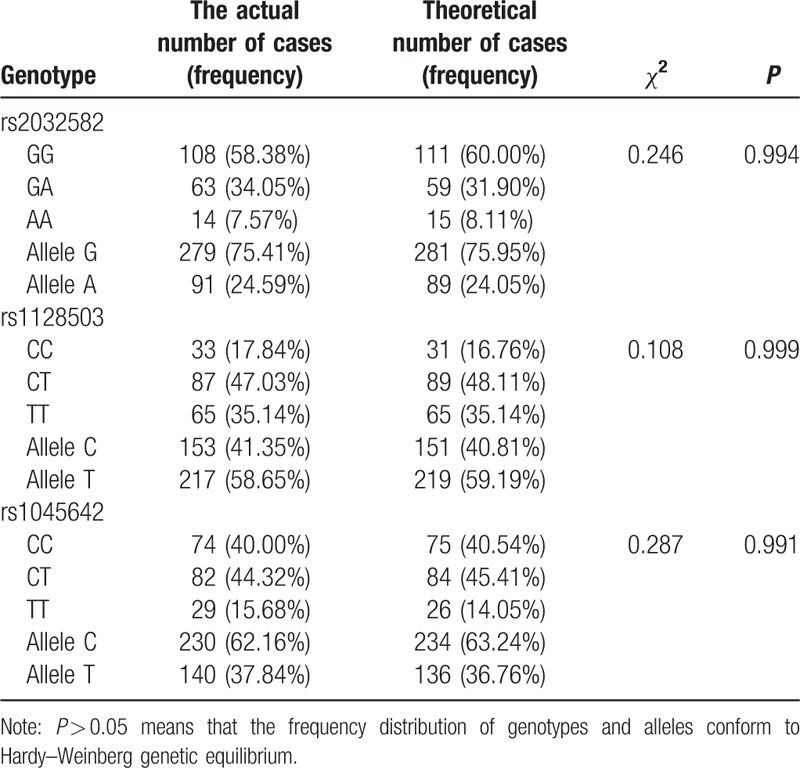
The frequency distribution of genotypes and alleles in *ABCB1* gene.

### 3.3 AS clinical indexes before and after etanercept treatment

Hypodermic injection of etanercept once a week was performed on 185 AS patients; among them, follow-up data were obtained from 168 cases after 12 weeks. Meanwhile, for 5.36% (9/168) of patients, symptoms they complained don’t respond to drug treatment; for 82.71% (139/168) of patients, symptoms moderatly respond to drug treatment; for 19.05% (32/168) of patients, symptoms signiificantly respond to drug treatment. The indexes including MS, function tests, erythrocyte sedimentation rate (ESR) or C-reactive protein (CRP) levels, BASDAI, and BASFI showed significant improvements after 12 weeks undergoing etanercept treatment (Table 3). In addition, the proportions of night pain, peripheral joint involvement, and the abnormal ESR or CRP were reduced. Importantly, the proportion of patients with BASDAI ≥4 was also significantly decreased compared with that before the treatment.

**Table 3:**
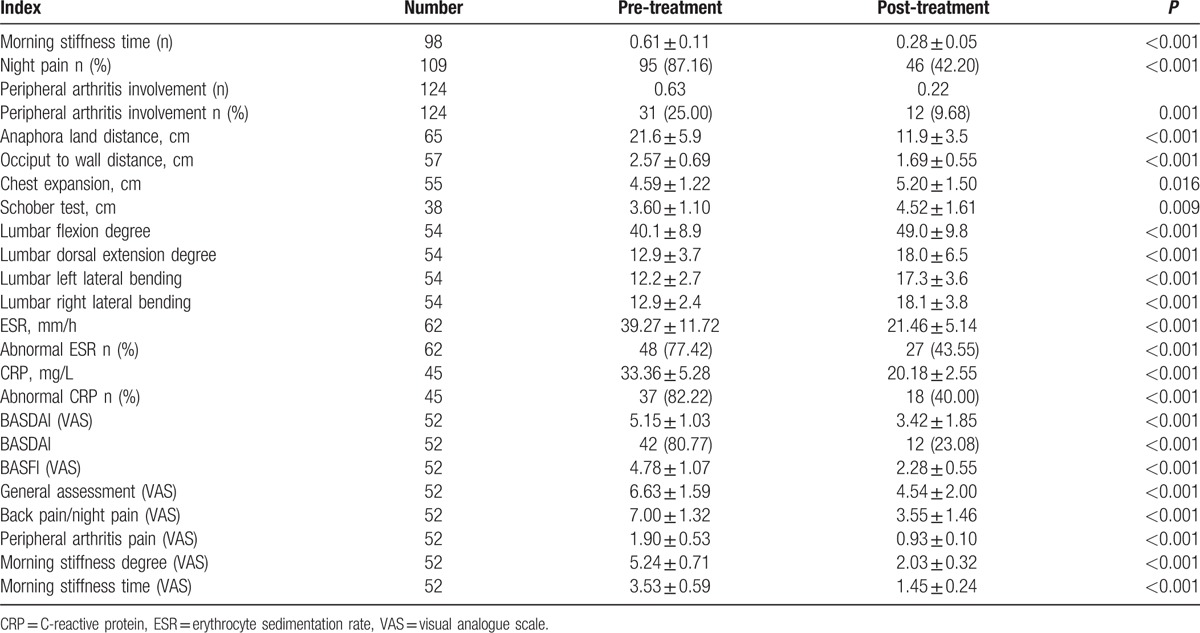
The index of 168 cases of AS patients before and after treatment.

### 3.4 BASDAI score of AS patients with different genotypes before and after etanercept treatment

After 12 weeks, the mean improvement values of A/A genotype (1.92 ± 2.11) was remarkably higher than G/G (1.41 ± 1.59) and G/A (1.45 ± 1.66) at rs2032582 (*P* < 0.05). The improvement ratio of A/A genotype regarding BASDAI50 was significantly higher than the G/G and G/A genotypes (all *P* < 0.05). No significant difference was found among 3 genotypes reaching the BASDAI20 but lowering BASDAI50 (*P* > 0.05). Also, the improvement ratio of A/A genotype that not reached BASDAI20 was lower than that in G/G and G/A genotypes (all *P* < 0.05). And, C/C genotype displayed increased mean improvement value (2.18 ± 1.66) in comparison with C/T (1.53 ± 1.38) and T/T (1.58 ± 1.30) at rs1128503 (all *P* < 0.05), and the same trends were revealed in the improvement ratio of patients reaching BASDAI20. The proportion of patients achieving BASDAI20 but lowering BASDAI50 also exhibited no statistical significance among the three genotypes (*P* > 0.05). The proportion of C/C patients with less than BASDAI20 was lower than CT and TT patients (all *P* < 0.05). There was no significant difference in mean BASDAI improvement value in the C/C, C/T, and T/T genotype at rs1045642 (*P* < 0.05). The proportion of patients reaching BASDAI50, patients reaching BASDAI20 but lowering BASDAI50, and patients lowering BASDAI20 had no significant difference among the 3 groups (*P* > 0.05) (Tables 4 and 5).

**Table 4:**
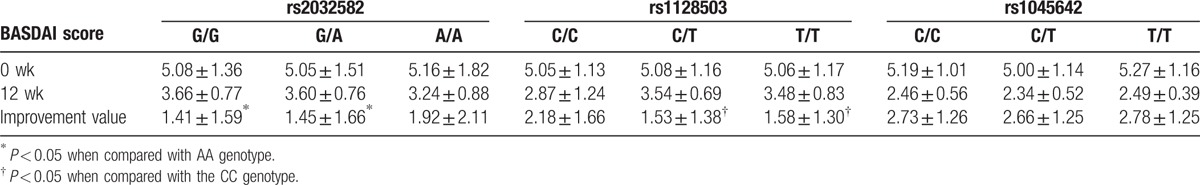
Comparison of BASDAI scores improvement values in AS patients with different genotypes after 12 weeks of etanercept treatment.

**Table 5:**
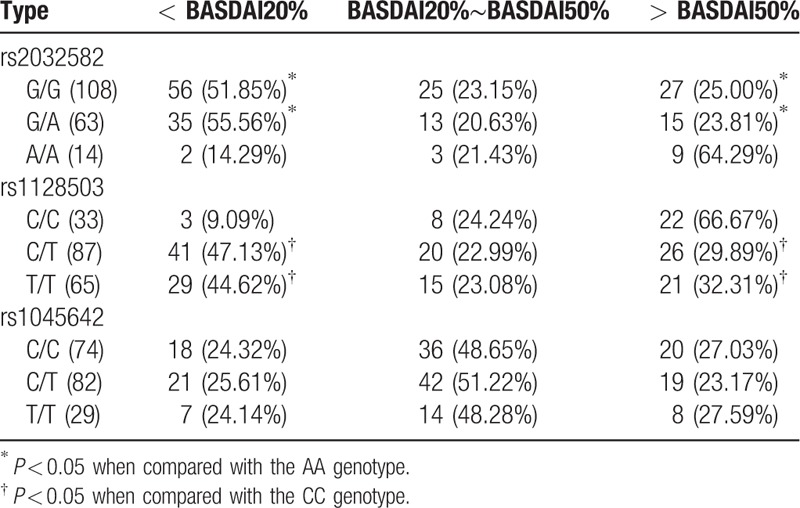
Comparison of the proportion of BASDAI scores improvement in AS patients with different genotypes after 12 weeks of etanercept treatment.

### 3.5 ASAS20/50/70 of AS patients with different genotypes after etanercept treatment

After 12 weeks, the proportions of G/G, G/A, and A/A patients who met the evaluation criteria ASAS20 at rs2032582 were 62.04%, 58.73%, and 92.86%, respectively. When compared with the G/G and G/A genotype, the proportion of A/A genotype reaching ASAS20 was significantly higher (*P* < 0.05). There was no statistical significance among G/G, G/A, and A/A patients of reaching ASAS50 and ASAS70 (*P* > 0.05). The proportion of patients reaching ASAS20 was 93.94% (C/C), 65.52% (C/T), and 64.62% (T/T) at rs1128503, indicating that the proportion of C/C was higher than C/T and T/T (*P* < 0.05). At the same time, no statistical difference was shown among C/C, C/T, and T/T patients reaching ASAS50 and ASAS70 (*P* > 0.05). The proportions of patients reaching ASAS20 were 66.22% (C/C), 64.64% (C/T), and 62.07% (T/T) at rs1045642 and no significant difference was observed among the 3 groups (*P* > 0.05). Consequently, the proportions of patients among 3 genotypes reaching ASAS50 and ASAS70 exhibited no statistically significant (both *P* > 0.05) (Table 6).

**Table 6:**
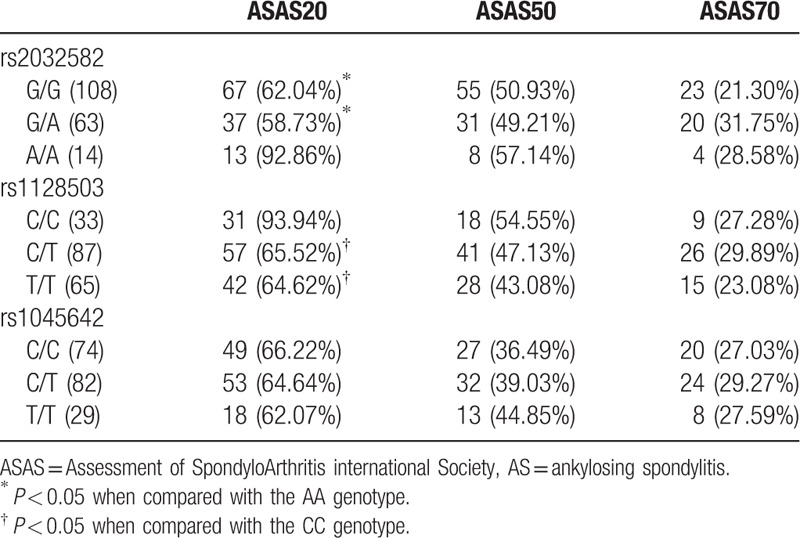
The ratios of AS patients with different genotypes reaching ASAS20/50/70 evaluation criteria after 12 weeks of etanercept treatment.

## 4 Discussion

Currently, a large amount of evidence has confirmed that most patients’ BASDAI scores were improved after etanercept therapy for AS. Given that it can effectively relieve the symptoms in AS patient, the etanercept was widely used in clinical treatment.^[8]^ However, there have been some differences in the efficacy of etanercept for different AS patients.^[9]^ Therefore, our study aims to investigate whether *ABCB1* genetic polymorphisms [rs2032582 (G2677T/A), rsll28503 (C1236T), rsl045642 (C3435T)] exert influences on drug metabolism and drug targets or drug receptor, leading to interindividual variability in drug disposition and efficacy of etanercept to reveal AS etiology and provide a potential approach for AS therapy.

Initially, our study showed that a significant improvement was observed in MS, functional tests, ESR or CRP levels, and BASDAI, BASFI scores after 12 weeks of etanercept treatment. Consistent with our results, Dougados et al^[21]^ reported that etanercept treatment contributed to rapid and systemic improvement in bone function and motion, and skeletal inflammation over 12 weeks in spondyloarthritis. In addition, our results reported that the mean improvement value of BASDAI score and the ratios of ASAS20 evaluation criteria in A/A genotype were significantly higher than that in G/G and G/A at rs2032582. Also, there was a significant improvement in mean improvement values of BASDAI score and the ratios of ASAS20 evaluation criteria in C/C when compared with G/G and G/A genotype at rs1128503. Therefore, all the results suggested that A/A and C/C genotypes improved the efficacy of etanercep. P-glycoprotein (P-gp), which was encoded by the *ABCB1* gene, was mainly expressed in the luminal surface of the blood–brain, blood–testis, and blood–ovarian barriers, and in the apical membranes of the kidney, liver, intestines, as well as adrenal glands.^[22]^ Furthermore, evidence is present for supporting the important role played by more gene polymorphisms in the function of P-gp via affecting activity and expression of P-gp, and by protecting organs from toxic xenobiotics caused by the energy-dependent efflux of substrates.^[23–25]^ Importantly, the possible mechanism of G2677T/A (Exon 21, rs2032582) on the impact of drug efficacy was that it contributed to the expression and function of P-gp by amino acid substitutions, resulting in improvement in the etanercept efficacy. At the same time, Wolking et al^[26]^ confirmed that polymorphisms and haplotypes of *ABCB1* have been closely correlated with changes in the disposition and response of drug, containing adverse events with multiple *ABCB1* substrates. Besides, C1236T in exon 12 is also other important polymorphisms of the gene, and it adjusted the protein folding as well as the speed and efficiency of translation by choosing alternative codons, lead to the promotion of etanercept efficacy.^[27–29]^

In addition, the study also found that there were no significant difference in BASDAI score and ASAS score at rs1045642 after 12 weeks, indicating that the genotypes of rs1045642 failed to affect etanercept efficacy on AS patients. The possible mechanism is that the amino acid sequence of C3435T (Exon 26, rs1045642) is not changed due to its silent mutations, so is P-gp activity. Furthermore, Menu et al^[30]^ demonstrated that the C3435T polymorphism was not associated with therapeutic response of antidepressants in naturalistic clinical conditions, which confirms the results of our results on efficacy. However, the study reported by Illmer et al^[31]^ shows a clearly increased relapse risk for acute myeloid leukemia patients with homozygous wild-type polymorphism of rs1045642. Thus, with regard to the impact of rs1045642 polymorphism on etanercept efficacy, we need further investigation.

In conclusion, this study reveals that *ABCB1* gene polymorphisms are involved in the etanercept efficacy on AS patients; thus, it can be a biological indicator of etanercept for individual patients with AS. However, there are also some limitations in this study, such as the influence of other factors, small sample size, and especially the lack of clinical staging and postoperative follow-up data of the selected cases. Thus, increasing sample and scientific methods are needed in future study.

## Acknowledgment

We would like to acknowledge the helpful comments on this paper received from our reviewers.
